# Methods to recognize work-related cancer in workplaces, the general population, and by experts in the clinic, a Norwegian experience

**DOI:** 10.1186/1745-6673-6-24

**Published:** 2011-09-07

**Authors:** Sverre Langård, Lukas Jyuhn-Hsiarn Lee

**Affiliations:** 1 Department of Occupational and Environmental Medicine, Oslo University Hospital, Oslo, Norway; 2 Division of Environmental Health and Occupational Medicine, National Health Research Institutes, Taiwan; 3 Institute of Occupational Medicine and Industrial Hygiene, College of Public Health, National Taiwan University, Taipei, Taiwan

## Abstract

**Background:**

In most countries, the numbers of work-related cancer identified are much lower than are the estimated total burden of cancer caused by exposure at work. Therefore, there is a great need to use all available practical as well as epidemiological methods for identification as well as to develop new methods of recognizing cases of work-related cancers.

**Methods:**

Primarily based on practical experiences from Norway, methods to identify cases of possible work-related cancers in the general population and at workplaces as well as methods to recognize more specific cases after referral to specialized clinics are reviewed in this publication.

**Results:**

Countries applying a number of the available methods to detect work-related cancer reach a reporting rate of 60 such cases per million, while other countries that do not employ such methods hardly identify any cases. As most subjects previously exposed to cancer causing agents and substances at work are gradually recruited out of work, methods should be versatile for identification of cases in the general population, as well as at work.

**Conclusions:**

Even in countries using a number of the available methods for identification, only a limited fraction of the real number of work-related cancer are notified to the labour inspectorate. Clinicians should be familiar with the methods and do the best to identify work-related cancer to serve prevention.

## Background

A number of estimates on the contribution from work exposure to the total burden of cancers in the world, as well as for specific countries, have been presented; the estimates vary from 2-3% to 6-7% [1-3]. Although these estimates vary greatly and no data are available from developing countries, we assume that the contribution by weight is about 5%. This gives an approximate number of yearly new weighed cancer cases (total burden) that are related to work exposure, for example, in Taiwan, of about 3,500-4,000 out of about 75,000 newly registered cases *per *year. As "total burden" means 100% contribution on case basis, and hardly any case is 100% caused by work exposure, the total number of new cases with causal contribution from work exposure is much higher. However, the fact remains that only a handful cases are recognized as work-related cancer in Taiwan.

Even in Western countries, for example, in Norway - with a total population of about 4.9 million and with about 80-years practice of identifying work-related diseases, including cancers - only about 300 cases of "suspected" work-related cancers are notified yearly to the work inspectorate. Assuming that 5% of the total burden of cancer is attributable from work-related exposures also in Norway, some 1,200-1,300 cases per year are due to work - out of about 25,000 incident cases. Underreporting is an obvious problem, even in developed countries like Norway, only about one-fourth of the total burden (300 out of 1,200) could have been reported as suspected cases. Currently, a smaller number, close to 200 (60~70%) out of the reported cases, are recognized by the Norwegian National Insurance Scheme (NIS) and private insurance companies as work-related to an extent that meets the requirements to be compensated as occupational diseases. However, since the number of reported cases is increasing, and since there is a delay in the handling of such cases by NIS, the real proportion of compensated cases may be a little higher.

A significant number of cancer cases are identified as work-related in most Western countries, Australia, Japan, Singapore and South Africa, but these numbers are far below the estimated real figures, as based on about 5% of the total cancer burden. In developing countries, only a few cases are reported each year. In countries like Thailand and Malaysia only a handful of mesotheliomas have been identified, and in Taiwan, less than 10 cases of mesothelioma (387 cases from 1979 to 2005 based on Taiwan Cancer Registry) have been authorized as occupational cancer [4]. Hardly any case of work-related cancer is identified and recognized in some developing countries.

The aim of this publication is to review methods applied in different countries to identify cases of possible work-related cancers in the general population and at workplaces, as well as methods to recognize more specific cases after referral to specialized clinics. Even in countries that apply a number of the methods for identification listed below, the rate of identification of cases reaches only a fraction of the estimated total number of work-related cases. Still, countries applying one, two or more of the available methods, appear to be more successful than are countries not applying any methods to identify cases. In this paper examples from Norway are used to the extent that they may be assume to help enhance detection and recognition of work-related cancers in other countries.

## Methods

Some of the methods listed below may identify work-related cases directly, while others may be considered as tools for identifying the presence of work-related cancers in the general populations at large or in small populations exposed to specific or possible carcinogenic agents, substances or compounds.

### I. Population-based methods to identify work- and environment-related cancers

#### a. Gender differences

Many countries have developed high quality cancer registers and/or mortality data, which may serve as tools to identify gender differences in the incidence or mortality rates for cancer locations common to the genders. In-depth analyses or epidemiological research of site specific cancers that occur with a higher incidence/mortality rate in males than in females can generally provide information as to why these differences occur. In the 1970's, male cancers of the nasal sinuses occurred with nine times higher incidence than in females in one Norwegian province [5]. The cause appeared to be that some 1,500 males in the province worked in a nickel refinery.

As for lung cancer, Finland may be an excellent example on male/female differences, with a very low incidence among females particularly in the 1950's - possibly close to the assumed archaic risk level for both genders - but also an unusually high incidence among men in the 1970's [6]. Although smoking is undoubtedly the most significant determinant for lung cancer in males in many countries, exposure at work may contribute about 15% of the cases by attribution in some countries [7].

#### b. Regional differences in the incidence or mortality

All countries with high quality data on cancer incidence and/or mortality may identify at least two-fold, and in some instances four- to six-fold, incidence/mortality for some cancer sites - in high risk versus low risk areas. For cancer sites with high rates of long-term survival, incidence data are more versatile for this purpose than are mortality data. Clearly, given that the figures are robust, such large differences cannot be explained by differences in the genetic shape or susceptibility.

In Norway two- to six-fold regional differences are observed for many cancer sites, i.e. for stomach cancer, cancer of the thyroid gland, malignant melanomas of the skin, and even for lung cancers [8]. Some of these large differences may be due to local causative factors, i.e. at a given factory or workplace where subjects have been exposed to given carcinogens. As mentioned before, such an example was observed in Norway in the 1960's and 1970's, where male cancer of the nasal sinuses was up to nine times higher in one province than in others, probably due to occupational exposure to nickel compounds in a nickel refinery [5].

Exposure factors in the general environment may also cause significant differences in the incidence of given cancer sites, e.g. regional differences for oral cancer in Taiwan, an endemic betel quid chewing area [9,10]. Some heavy metals contaminated in the soil might promote oral cancer development in local residents [11].

#### c. Epidemiological studies on work-related cancers

Whenever large differences in incidence or mortality are observed, one should initiate appropriate epidemiological studies among the general population of the areas/municipalities that present high incidence or mortality of site-specific cancer, in order to identify work-related contributions to the differences. In some instances one may already have a hunch as to what the possible causation is, e.g. given workplaces in the region. If that is the case, it may be more versatile to carry out epidemiological studies among the known exposed population, thus identifying accurately - for previous and current workers - their previous exposure in that workplace and previous workplace(s), subsequently carrying out a historical, prospective cohort study. Having identified that cohort and the participant's exposures, linkage to cancer incidence or mortality data may be carried out. A major site of environmental pollution may also be the cause of local enhancements of cancer incidence/mortality.

In Norway, only a handful of work-related cancers were reported to the work inspectorate in the 1950's and 1960's. It was only after epidemiological studies on work-related cancers were performed from the early 1970's [12,13] and onwards, that physicians, workers, worker's unions, and also the news media and the public, began paying attention to the carcinogenic hazards in workplaces. Once these first studies were published, a wave of new cancer-studies in different industries and workplaces were initiated and carried out during the second half of the 1970's and the early 1980's [14-19]. Once the results from the different studies had been presented to the workers involved, and subsequently appeared in the scientific journals, an increasing number of suspected cases of work-related cancer were identified and reported by physicians to the work inspectorate as well as being referred to the clinical departments of occupational medicine. While presenting this information to workers, the physician scientists also frequently appeared in the mass media to inform the public about work-related cancers. More frequent referral of patients to the clinical departments clearly appeared to be related to the awareness about work-related cancer among the physicians in the country.

Thus, irrespective of local or regional differences in the incidence or mortality for specific cancer sites in the general population, epidemiological studies on the incidence or mortality of cancer should be carried out among industrial workers and also among workers of other workplaces whenever there is science-based indication of an existing carcinogenic hazard in a workplace. The experience from the Nordic countries clearly shows that performing cancer studies among workers exposed to carcinogenic agents or compounds strongly enhances the awareness in work-related cancers among medical professionals and workers.

#### d. Specialized clinical departments for work-related diseases

The establishment of specialized hospital-based departments/clinics for occupational medicine was initiated in Norway as of 1977. Subsequently, five more clinics were established during the second half of the 1980's and early 1990's. Later, two clinics have merged, thus leaving five clinics for occupational and environmental medicine in Norway today. Only a few cases of work-related cancer were identified before these departments were established. These clinics also have carried out many studies on work-related cancer.

Research activities, as well as the clinical work carried out by these clinics, have contributed to enhancement of regional interest on work-related cancers/diseases, particularly among primary health care physicians and occupational health physicians. The *awareness *among these primary health physicians on cancers possibly being work-related strongly depends on their *relevant education*. Primary health physicians' education on work-related cancers/diseases relies on the following fundamental factors:

a) their primary education in medical school; and

b) continuous updating/courses during the physician's whole career, to retain and enhance their knowledge on as well as alertness of identifying work-related cancers/diseases.

One of the major tasks of these clinical departments is to facilitate education on work-related cancers/diseases among primary health care doctors as well as occupational health physicians, e.g. by arranging relevant courses as well as lectures among colleagues. The clinical departments also have succeeded in gently "infiltrating" other specialty courses with lectures on the occurrence of and necessity of awareness of work-related cancers/diseases. In Norway, attempts to introduce occupational medicine beyond basic education in the curriculum of the medical schools have generally been less successful. However, medical schools of the other Nordic countries have been more willing to widen the scope of education on occupational medicine and on work-related cancer in medical schools.

Also, regional and national education of physicians on this field has contributed to enhancement of the *awareness *among *workers *that their cancer/illness may be related to work exposure. The clinical departments have also facilitated awareness among the workers that new cases of cancer may be work-related, and have been active in informing the public and workers on the results of cancer studies and the possibilities of work-related cancers. Worker's unions may to a great extent support enhancement of the awareness among workers that some cancer cases may be caused by exposure at work.

#### e. Obligatory reporting by all physicians on *suspected *cases

Physicians must file reports on suspected cases of work-related cancer to the work inspectorate in some countries whenever the physician suspects that the disease may be related to work exposure. In Norway, notification on work-related diseases started in 1933 and primarily to report cases of silicosis to the work inspectorate, which had a high incidence in the 1930's [20]. The primary purpose of this reporting system was to enhance prevention by notifying the work inspectorate on hazardous workplaces that might cause occupational diseases, thus giving the inspectorate the opportunity to visit the worksites of concern and to subsequently communicate measures to the employers and employees on preventive strategies.

A form for reporting meant for physicians to notify on work-related cancers/diseases, should be characterized by "low threshold". The reporting form must be simple in order to ensure a low threshold. The physician should only be required to provide the patient's identity, suspected relevant exposure, the identity of the employer where the suspected exposure took place, and the physician's judgment as to possible work-relatedness. The physician should not be required to attempt to "prove" that the cancer/disease is work-related. The reporting should only be based on *suspicion *of work-relatedness. Reporting on "suspected" work-related cancers/diseases should be obligatory, thus avoiding concerns that the physician filing a report on work-related cancer could be accused of trying to hurt the workplace of concern.

In Norway only a handful of work-related cancers, e.g. a few mesotheliomas and nickel-related cancers, were notified to the labor inspectorate prior to the 1970's. Without the legislative support to make report filing obligatory, lawsuit could hypothetically be filed against the physician for trying to hurt a workplace. There should also be a reward to the physician for reporting, corresponding to the average time she/he spends on compiling necessary information on exposure, filling the form and filing the report. Informed consent should be obtained prior to filing report.

Once the report form is filed, it must be up to the labor inspectorate, the insurance scheme and/or specialists in occupational medicine, to clarify the work-relatedness of the cancer case or disease and to determine to what extent the case is work-related.

To motivate report filling, it is of major importance that the physician receives feedback that reporting cases serves prevention and also may have positive consequences for the patients. The feedback system must make it clear to the physicians that reporting is of great significance for both aspects. Today, an online system for reporting and feedback can easily be introduced on the statistics of such reports, the uses of the filed reports for prevention, and the patient benefits of the reports. Such feedback appears to enhance the number of filed reports.

There are, however, still major deficiencies in filing reports on work-related cancer also in many Western countries, e.g. many physicians are not filing reports, indicating a significant potential for improvements in the reporting system. With obligatory reporting, there may be a potential that a physician failing to file a report an obvious case of work-related cancer could be accused of malpractice for not reporting relevant cases.

In the mid 1980's an alteration was made in the filing system in Norway; an additional copy of the reporting form was to be filed to the National Insurance Scheme (NIS), leaving the responsibility for case follow-up to the insurance scheme, e.g. to refer the case subject to a clinical department of occupational and environmental medicine to have the work-relatedness scrutinized. Based on a comprehensive identification and quantification of previous exposure, as well as consulting relevant scientific literature, the department physician subsequently files an expert statement to the insurance scheme. This statement serves as basis for judging work-relatedness for the insurance, which makes the decision on whether or not the exposure and the disease justifies acceptance as occupational disease/cancer, in accord with current legislation.

As of today about 300 cases of cancer are reported yearly to the labor inspectorate in Norway, of which about two thirds are cancers of the airways - including mesotheliomas.

#### f. Tool for detection of cases based on cancer or mortality registers

Resulting from an incident in the mid 1980's, in which the "Data inspectorate" - referring to patients' confidentiality - denied filing reports on work-related cancers for deceased subjects that had been identified in an epidemiological study [21]. As it was felt to be inappropriate that some cancer case subjects in a given cohort could be scrutinized for possible compensation for occupational cancer, while others were denied such scrutiny, a letter was drafted to - with permission from the Data inspectorate - be sent from the Cancer Registry to patients with certain cancer diagnoses, known frequently to be work-related. To select case subjects to receive such a letter, the case subject was linked to two or three censuses, which contain information on occupations. The patients to receive the letter are those who have one of those cancer diagnoses and certain high-risk job titles - over two or three consecutive censuses - that are known to carry an elevated risk of work-related cancers. The letter informs the patient that his/her cancer might be work-related, and suggests referral by the primary physician to a clinical department of occupational and environmental medicine to for scrutiny on possible work-relatedness. Currently such letters are submitted to patients in some countries, e.g. Norway and Canada.

#### g. Educating colleagues with specialties frequently encountering cases of work-related cancers

As a large proportion of the known work-related cancers occur in the respiratory organs, there should be ways to enhance the awareness of the presence of work-related cancer among specialists frequently encountering nasal sinus cancers, mesotheliomas, and different types of lung cancers. A method for asbestos-related cancers is simply to convince pulmonologists, radiologists, internists, and pathologists to refer all case subjects with both lung cancer and typical asbestos-related pleural plaques or calcifications to the departments of occupational and environmental medicine. The same should be done for all malignant mesotheliomas, of which nearly 95% has been attributable to previous exposure to asbestos fibers [2]. Furthermore, case subjects with typical asbestos-associated pleural plaques and a cancer of other organs generally known be related to past exposure to asbestos, e.g. cancer of the oesophagus, colon and rectums, as well as of the kidneys and the urinary bladder [22-24], should be referred for scrutiny on work-relatedness.

#### h. Screening the general population by questionnaires

Workers previously exposed to asbestos fibres and other carcinogenic agents or compounds in workplaces eventually end up in the general population. Consequently, most subjects previously exposed to work-related carcinogenic factors are found in the general population. Identification of these previously exposed subjects can be accomplished by designing questionnaires to be submitted to certain age groups, e.g. preferably males aged 40 years and above or more than 50 years. This transfer of disease risks from the workplace to the general population has been particularly strong for the subjects previously exposed to asbestos for which the seized use of asbestos over the past 3 decades has enhanced the transfer of asbestos-exposed subjects at the work site to the general population. Consequently, questionnaires designed to identify previous workplaces and work-related exposure to asbestos, should be designed for screening of the general populations. To account for latency, questions should be developed specifically to identify previous asbestos-exposure in the industry/workplaces of the region more than 20 years ago.

Such population-based exposure screening might be combined with various other types of screening instruments, specifically designed to identify certain cancers or exposure markers. In the early 1980's, a large scale screening in which 21,453 males, aged 40 years and above, were screened by questionnaires and lung X-rays was carried out in Norway, indicating that population-based screening was indeed efficient [25]. 3,888 were confirmed exposed and 2,820 had uncertain exposure to asbestos, of whom 470 (2.2%) had asbestos-related lung disorders, including 86 parenchymal asbestoses. Questionnaire screening, possibly combined with lung X-rays, could be carried out in the neighborhoods of previous work sites with a known high probability of exposure to asbestos or other carcinogenic agents.

### II. Clinical methods to recognize work- and environment-related cancers

Some cancer cases are easily recognized as work-related, e.g. near 95% of *malignant mesothelioma *cases are caused by exposure to asbestos [2]. Close to all 60-65 yearly new male cases [8] in Norway occur in the previously asbestos-exposed subjects [7]. Therefore, once exposure to asbestos is documented in cases of *malignant mesothelioma*, the insurance scheme accepts the cases of malignant mesothelioma as being subject to compensation.

Except for some exposure factors with a skewed distribution towards a given histological type, e.g. adenocarcinoma of the lung after asbestos exposure [26], work-related cancers are histologically not distinguishable from non-work-related cases. Thus, distinguishing a work-related cancer case from a non work-related case can only be accomplished through a very comprehensive exposure history.

#### a. Work- and exposure history

The only way to identify the possible causes of a given case of cancer is to identify and to (semi)quantify work- and environmental exposure in the past, preferably from the conception onwards - up to the day the case subject is referred for causality determination. Recognition of work-relatedness of cases is fully dependent on a comprehensive and precise life-long exposure history, documenting *all relevant exposure factors during all periods *of previous work, subsequently *(semi)quantifying *all the exposures that possibly may have contributed to increased risk of the cancer cases of concern.

##### Work- and exposure history

It is recommended to separate the anamnesis in a section for chronological employment work history and one for specific exposure during each employment period.

The *general employment history *should contain dates for start and for quitting for each consecutive job.

The section for *specific exposure *should identify all exposure factors as far as possible in order to quantify both levels of exposure and the duration of exposure to each significant exposure factors during every employment period.

As *example*, if a patient had welded in a shipyard from February 1, 1959 until December 31, 1968, information on the main types of welding that he carried out should be identified specifically as well as the duration of each type of welding operation, the extent to which he welded in confined spaces and the extent to which he welded in the work-shop, the types of steel that he welded, the types of electrodes he used in each operation, the extent to which there were other welders in the neighborhood, if there was ongoing concomitant insulation (asbestos), the extent to which he use certain kinds of respirators, and finally - the extent to which there were other possible exposures, i.e. soldering.

Furthermore, it should be determined whether or not the data from environmental monitoring relevant for the patient is available for his workplaces and whether or not that data is retrievable. The extent to which the work hours were distributed between each of the different tasks should also be recorded. Moreover, the size and space of the work facility in which the employee worked and the possibility of additional bystander exposure should be considered. Extended periods of absence from work should also be registered. A description of the facility and the presence of general and/or local ventilation should be taken into account.

Some countries have compiled measurements in industries in national databases for current and historical measurements on exposure in its workplaces, which is a useful source of information on levels of exposure. The databases may contain measurements in the workplace of concern or in comparable workplaces from the same time period.

When the work/exposure history is completed, the information on the subject's exposure to various substances, dusts, agents, or substances, must be in depth, thus permitting judgment - preferably quantification - as to the subject's exposure-related current and projected (future) added risk of one or a number of cancers. (See below on reference articles).

##### Significance of the "latent period"

Due to the long latent period - generally defined as the time period between the first exposure to the causative agent/compound and the occurrence/diagnosis of the disease - for most work-related cancers, exposure in early life and early work periods are more likely to have contributed as a cause for the disease than exposure late in the work period. Consequently, it is of major importance to compile an accurate exposure history for the early employment periods. Therefore, when searching either cohort- or case-referent scientific articles for reference, one should search for publications that account for latency or that present the data in a way that permits physicians to apply the dose-response data and account for latency at the same time.

##### Reference articles

High quality scientific articles that present robust results and deal with exposure and cancer outcome closely matching those of the case subjects, are obligatory in order to estimate and/or judge the (semi-)quantitative risk of cancer in the case patient prior to occurrence of the disease. Reference articles that closely match the patient's exposure as well as the cancer site should be preferred. Articles that report on data from a well designed and properly performed epidemiological studies, also presenting dose-response relationships for the exposure-factor(s) and the cancer outcome of concern, should also be preferred. To be applicable, the dose-response scale presented in the publication should include the patient's exposure level in terms of intensity and duration, preferably during a time period that adheres to the experienced "latency" period for the cancer of concern.

Such reference articles could preferably be common to all the medical professionals who work in a clinical department of occupational and environmental medicine. The articles may prove versatile to permit individual assessment of disease risk(s). There could be common or "standard" reference articles for the most frequent cause/effect relationships that the department is scrutinizing. These reference articles should be updated as soon as new and possibly more representative articles are published.

In addition to sets of common reference articles on frequently encountered possible causal relationships, additional literature should be reviewed for individual patients in order to find published exposure situations that match the patient's exposure and disease even better than the cases that can be found in the sets of common reference articles.

##### Other contributing causes

Once a complete exposure history on relevant work- and environment-related exposures has been compiled for the patient, one is in the position to account for these exposures such as cigarette smoking, use of ethanol, passive smoking during childhood, at work and at home, exposure to radon daughters at work or at home, and other possible competing causes of the disease case.

##### Quantifying individual disease risks

By compiling comprehensive information on all the past significant exposure factors, that are known to carry an intrinsic potential to increase individual risks of cancers or other diseases, it may be possible to quantify the subject's risk of the cancer or disease of concern as well as for other cancers, prior to occurrence/detection of the case. Based on the compiled exposure-information, the subjects' individual - current and projected - levels of risks for different cancers can be estimated by consulting robust scientific literature that represents dose-response data for the exposure-factor(s) of the patient. Based on the subjects' exposure, current and projected cancer and disease risks may be derived from the dose-response information in such high quality epidemiological data. Accumulated cancer/disease-risk related to the most significant disease determinants of a subject does not tell anything about causation as defined by Rothman et al [27]. However, until real quantitative measures of contributory cause are developed, accumulated exposure-related risk may possibly serve as a surrogate for causal contribution.

##### Sufficient exposure

Whenever all the above information on exposure is compiled - which is necessary in order to judge an individual case of cancer for work-relatedness - one has to judge whether the prime exposure alone, or in combination with other exposure factors, contributes sufficient exposure to increase the risk of cancer sufficiently to be considered to have caused the case - alone or in combination with the other work- or non-work-related exposure factors. Whether or not sufficient exposure might be considered to have accumulated during a relevant time period should be based on dose-response data in representative scientific literature. Experienced or assumed latent period - as based on data from the scientific literature - also has to be accounted for in this judgment, e.g. 15-25 years or more, depending on which cancer has occurred, the intensity of exposure, and the potency of the causal factors(s).

##### Doubling of the risk

**Figure 1:**
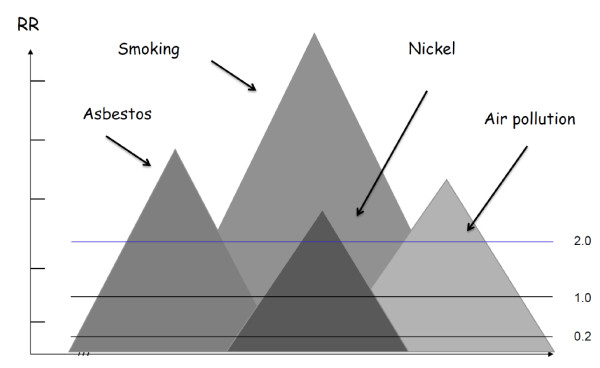
**Identify doubling of the risk for lung cancer**. Insurance schemes and companies in some countries apply the notion "doubling of the risk" of a given case of exposure-related cancer as criterion to compensate work-related cancers. Any population may comprise a number of subpopulations with cancer risk related to a number of exposure factors as illustrated. As illustrated, a study may have identified just in excess of doubling of the lung cancer risk in a small sub-population of those exposed to nickel compounds when referring to the age-adjusted general male population. Had the archaic risk, here presumed to be at 0,2 compared to 1,0 for the general population, the **relative risk (RR) **in the same sub-population of the nickel-exposed would have been at more than 10,0 in the reference level. Which background risk should serve as reference for "doubling"?.

could serve as criterion of insurance companies for accepting a cancer case as an occupational disease. The degree of increased association with a specific exposure is determined usually with measures such as relative risk or absolute risk. Basically the stronger the association, the less likely it is due to error. National insurance schemes and insurance companies in some countries apply the notion "doubling of the risk" for a given case of exposure-related cancer as a basis for accepting the case as work-related, hence for compensating the cancer as occupational disease. Many weaknesses of the notion *doubling of the risk *were discussed in depth by Greenland [28] and Morfeld [29]. In this paper we add another weakness by pointing to the lack of defining what *doubling of the risk *is based on, i.e. what is the reference for doubling. When no level of risk is defined to serve as basis for doubling, the notion is non-informative, hence a "floating unity" (Figure 1). Therefore, to avoid the problem of referring to *doubling of the risk *in expert-statements to an insurance schemes or companies, one should preferably present the estimated *á priori *absolute risk of the patient prior to the occurrence of the disease, along with the risk for *two different reference populations*;

a) the *absolute risk *in age- and gender-specific risk of the *general population*, and

b) the *presumed archaic risk *of the cancer or disease that corresponds to the subject's age/gender.

An approximate archaic risk may be estimated for a number of cancer sites, i.e. lung cancer in Finland - where the incidence in women in the 1950 was about 2 cases *per *100,000 *per *year [6]. The use of these two alternative entities as reference may result in differences in the attribution to different causes. If one is not in the position to present both these two sets of preferred reference-risks, the assumed most appropriate level of reference risk should be identified and also defining this risk level in absolute terms.

Some insurance schemes or insurance companies may demand estimates of the weighted attribution to different contributing exposure factors. Such attribution - or partition - of weighted causality to the identified causal factors could be based on comparison of the cumulated exposure-related risks of the cancer of concern, resulting from exposure to different exposure factors. Attributed weighted contribution to the different identified causal factors could be based on the risk of the cancer of concern that has been accumulated resulting from each individual exposure factor prior to occurrence of the cancer case [7]. To permit such an estimate, cumulative exposure to the causal factors in relevant time windows must be accurately compiled in order to permit comparison with dose-response data on the association of concern acquired from one or more robust, well designed and performed epidemiological study/ies. To allow calculation of cumulated relative or absolute risk of the cancer of concern in the case subject, the level of absolute risk of the reference population must be identified [23].

As criteria are not defined for the terms "robust and well designed" reference studies, these terms may be ambiguous. Robustness implies adequate power of the study and well designed implies that exposure to all significant exposure factors have been compiled at least for the majority of the participants - preferably for nearly the whole study population - permitting analysis of synergetic effects as well as statistical interactions in accord with suggestions by Greenland et al [30].

No comparison can be made of the patient's *á priori *cancer risk to the "background" risk (reference risk) if the reference risk is undefined. One possible reference risk is that of the gender and age adjusted general population. Another reference risk could be the archaic risk of cancer, e.g. the estimated or assumed age and gender-adjusted risk of cancer in the (hypothetical) absence of all cancer causing factors in the environment and at work. The archaic risk is not known for many cancer sites, but can be estimated by consulting the lowest ever incidence of a specific site in countries with high quality incidence data.

As tobacco smoking is a strong determinant of many different cancers, in particular for cancer of the respiratory organs, smoking is frequently a competing cause for cases of cancer that are partially caused by exposure at work. As lung cancer is the cancer site with highest incidence of work-relatedness in the Western world, and asbestos is the work-related cause with the highest incidence, asbestos exposure and tobacco smoking are commonly combined causes of lung cancer cases. Hence, if the insurance company/scheme asks for weighed attribution to the cancer case by smoking and asbestos, respectively, one may attribute in accord with the following suggestion [31].

## Discussion

Expert statements on work-relatedness should consider solely whether or not - and to what extent - the exposure(s) of concern is/are likely cause(s) of the cancer/disease. The statement should be based on robust scientific literature that reflects the exposure of the case subject as closely as possible, which is aimed at in Norway. Based on long term experience from clinical handling of case subjects, we advice that expert statements should preferably be based exclusively on scientific evidence. However, as robust scientific literature that represents the exposure and the disease of the subject is not always available, the medical expert may be left with an absence of relevant reference literature, leaving sound judgment based on experience as the only option. Whenever that is the situation, the expert should not attempt to provide science-based responses to the insurance scheme/company's specific questions, because that is impossible. Instead, the expert should clearly state that no relevant scientific literature is available to support a science-based statement. She/he could also state specifically the level of confidence in her/his responds to the different questions.

Whether a cancer case or disease is to be considered as an "occupational disease", hence to be compensated, is not to be judged by the medical expert filing a science-based statement to an insurance scheme or company. That decision should exclusively be taken on the basis of the rules for decision-making to which the insurance scheme or company must adhere. The insurance scheme/company is likely to ask questions on causality and possibly on weighed attribution in order to get science-based answers that may meet their needs, permitting judgment on the basis of their roles for acceptance as "occupational disease".

If the rules for acceptance of the insurance scheme or company consider doubling of the risk as sufficient enough to judge the case as occupational cancer/disease, one should attempt to clarify the weaknesses of that notion, as suggested above. One should show how "doubling" gives different results for attribution depending on which reference level that is used.

No one is able to tell which factor that initiated the development of a given case of cancer whenever the case subject has been exposed to two or more factors that may have initiated the cancer. Thus, it might be argued that attribution of relative weight of causation on the basis of relative level of estimated cancer risk prior to occurrence of the cancer may not be more appropriate than just guessing the contribution from each causative factor. On the other hand, when having applied this method in, e.g. 100 similar cases, one may be quite sure that the average outcome result becomes much closer to the true weights of attributed causation than the use of other methods of attribution can demonstrate. The method [31] also permits to account for synergetic interactions, e.g. enabling attribution of the interaction effects of smoking and asbestos in the development of lung cancers. Also, whenever the contribution by asbestos to the total cancer risk is relatively high in a given case, attribution of the outcome contribution resulting from the *á priori *risk from the effect of interaction in proportion to the relative risk contribution of each of the two factors, the attribution to asbestos frequently becomes higher than when applying the notion "doubling of the risk" as criterion for acceptance as "occupational disease".

Another consequence of attribution in accord with the *á priori *exposure-related risk is that, whenever the case subject who has contracted lung cancer - receives full workers' compensation on the basis of doubling or more of the lung cancer risk related to exposure to asbestos, it may become difficult to seek alternative compensation for other causative factors which might have elevated the *a priori *risk 10-20 times above the same background level, i.e. tobacco smoking.

## Conclusions

Although it seems unlikely ever to accomplish complete identification and reporting of work-related cancers, even when applying all available methods of identification, countries applying these methods, e.g. Norway, can demonstrate a much higher rate of identification of cases than countries not using the methods. Also, a country like Japan, which identified only 50-60 yearly cases of work-related cancer up to 5-6 years ago, and has currently increased that number to 2-3,000 over the last few years due to asbestos-associated cancers [32]. The figures for increased rate of detection in Japan clearly shows that other countries in Asia have a huge potential of identifying large numbers of work-related cancers, hence hopefully giving them the ability to prevent such cancer cases in the future.

## Competing interests

The authors declare that they have no competing interests.

## Authors' contributions

SL constructed the design and drafted the manuscript. LJHL participated in the design and collected the data, and revised the manuscript critically. All authors read and approved the final manuscript.

## References

[B1] Doll R Peto R The causes of cancer: quantitative estimates of avoidable risks of cancer in the United States today J Natl Cancer Inst 1981 66 1191 1308 7017215

[B2] Rushton L Hutchings S Brown T The burden of cancer at work: estimation as the first step to prevention Occup Environ Med 2008 65 789 800 10.1136/oem.2007.037002 18079154

[B3] Rushton L Bagga S Bevan R Brown TP Cherrie JW Holmes P Fortunato L Slack R Van Tongeren M Young C Hutchings SJ Occupation and cancer in Britain Br J Cancer 2010 102 1428 1437 10.1038/sj.bjc.6605637 20424618 PMC2865752

[B4] Lee LJ Chang YY Wang JD Impact of malignant mesothelioma in Taiwan: a 27-year review of population-based cancer registry data Lung Cancer 2010 68 16 19 10.1016/j.lungcan.2009.05.016 19535165

[B5] Norway Cancer in Norway 2008 Cancer Registry of Norway

[B6] The NORDCAN project http://www-dep.iarc.fr/NORDCAN/english/frame.asp

[B7] Langard S Prevention of lung cancer through the use of knowledge on asbestos and other work-related causes--Norwegian experiences Scand J Work Environ Health 1994 20 Spec No 100 107 7846485

[B8] Bray F Cancer in Norway 2006: Cancer Incidence, Mortality and Prevalence in Norway. Oslo, Norway: Cancer Registry of Norway 2006

[B9] Parkin DM Bray F Ferlay J Pisani P Global cancer statistics, 2002 CA Cancer J Clin 2005 55 74 108 10.3322/canjclin.55.2.74 15761078

[B10] Chen YJ Chang JT Liao CT Wang HM Yen TC Chiu CC Lu YC Li HF Cheng AJ Head and neck cancer in the betel quid chewing area: recent advances in molecular carcinogenesis Cancer Sci 2008 99 1507 1514 10.1111/j.1349-7006.2008.00863.x 18754860 PMC11159516

[B11] Chiang CT Lian Ie B Su CC Tsai KY Lin YP Chang TK Spatiotemporal trends in oral cancer mortality and potential risks associated with heavy metal content in Taiwan soil Int J Environ Res Public Health 2010 7 3916 3928 10.3390/ijerph7113916 21139868 PMC2996216

[B12] Pedersen E Hogetveit AC Andersen A Cancer of respiratory organs among workers at a nickel refinery in Norway Int J Cancer 1973 12 32 41 10.1002/ijc.2910120104 4790709

[B13] Langard S Norseth T A cohort study of bronchial carcinomas in workers producing chromate pigments Br J Ind Med 1975 32 62 65 164880 10.1136/oem.32.1.62 PMC1008024

[B14] Langard S Andersen A Gylseth B Incidence of cancer among ferrochromium and ferrosilicon workers Br J Ind Med 1980 37 114 120 7426461 10.1136/oem.37.2.114 PMC1008677

[B15] Hilt B Rosenberg J Langard S Occurrence of cancer in a small cohort of asbestos-exposed workers Scand J Work Environ Health 1981 7 185 189 20120583 10.5271/sjweh.3110

[B16] Kjuus H Lislerud A Lyngdal PT Omland H Stave O Langard S Cancer and polluted work places: a case-control study Int Arch Occup Environ Health 1982 49 281 292 10.1007/BF00377937 7068240

[B17] Magnus K Andersen A Hogetveit AC Cancer of respiratory organs among workers at a nickel refinery in Norway Int J Cancer 1982 30 681 685 10.1002/ijc.2910300602 7160938

[B18] Baksaas I Lund E Skjerven JE Langard S Vellar OD Aaro LE [Cancer in merchant seamen. A group study] Tidsskr Nor Laegeforen 1983 103 2317 2320 6665779

[B19] Heldaas SS Langard SL Andersen A Incidence of cancer among vinyl chloride and polyvinyl chloride workers Br J Ind Med 1984 41 25 30 6691932 10.1136/oem.41.1.25 PMC1009231

[B20] Evang K Silicosis in Norway. [Silicosis pulmonum i Norge] Tidsskr Nor Lægeforen 1937 22 1174 1186

[B21] Solli HM Andersen A Stranden E Langard S Cancer incidence among workers exposed to radon and thoron daughters at a niobium mine Scand J Work Environ Health 1985 11 7 13 2986282 10.5271/sjweh.2261

[B22] Selikoff IJ Hammond EC Churg J Asbestos exposure, smoking, and neoplasia JAMA 1968 204 106 112 10.1001/jama.204.2.106 5694532

[B23] Enterline PE Hartley J Henderson V Asbestos and cancer: a cohort followed up to death Br J Ind Med 1987 44 396 401 3606968 10.1136/oem.44.6.396 PMC1007840

[B24] Selikoff IJ Hammond EC Seidman H Mortality experience of insulation workers in the United States and Canada, 1943--1976 Ann N Y Acad Sci 1979 330 91 116 10.1111/j.1749-6632.1979.tb18711.x 294225

[B25] Hilt B Lien JT Lund-Larsen PG Lund K Langard S Asbestos-related findings in chest radiographs of the male population of the county of Telemark, Norway--a cross-sectional study Scand J Work Environ Health 1986 12 567 573 3493527 10.5271/sjweh.2105

[B26] Lee BW Wain JC Kelsey KT Wiencke JK Christiani DC Association between diet and lung cancer location Am J Respir Crit Care Med 1998 158 1197 1203 9769282 10.1164/ajrccm.158.4.9804089

[B27] Rothman KJ Greenland S Poole C Lash TL Rothman KJ, Greenland S, Lash TL Causation and Causal Inference Modern epidemiology 2008 3 Philadelphia: Wolters Kluwer Health/Lippincott Williams & Wilkins 5 31

[B28] Greenland S Relation of probability of causation to relative risk and doubling dose: a methodologic error that has become a social problem Am J Public Health 1999 89 1166 1169 10.2105/AJPH.89.8.1166 10432900 PMC1508676

[B29] Morfeld P Years of Life Lost due to exposure: Causal concepts and empirical shortcomings Epidemiol Perspect Innov 2004 1 5 10.1186/1742-5573-1-5 15601477 PMC545055

[B30] Greenland S Lash TL Rothman KJ Rothman KJ, Greenland S, Lash TL Concepts of Interaction Modern epidemiology 2008 3 Philadelphia: Wolters Kluwer Health/Lippincott Williams & Wilkins 71 86

[B31] Langard S Attribution of causal weight by work- and environment-related diseases based on epidemiological data. [Fordeling av årsaksvekt ved arbeids- og miljøbetingede sykdommer på basis av epidemiologiske data.] Nor J Epidemiol 1994 26 31

[B32] Furuya S Asbestos campaign: Update on the recent development ANROAV 2007 Meeting 2007

